# First report of *Angiostrongylus vasorum* in an African golden wolf (*Canis lupaster*) in Algeria

**DOI:** 10.1186/s13071-024-06534-9

**Published:** 2024-10-28

**Authors:** Noureddine Mechouck, Georgiana Deak, Angela Monica Ionică, Corina Toma, Andrada Gabriela Negoescu, Marian Taulescu, Zihad Bouslama, Andrei Daniel Mihalca

**Affiliations:** 1 Department of Parasitology and Parasitic Diseases, University of Agricultural Sciences and Veterinary Medicine of Cluj-Napoca, 3-5 Calea Mănătur, 400372 Cluj-Napoca, Romania; 2 Molecular Diagnosis Laboratory, Clinical Hospital of Infectious Diseases of Cluj-Napoca, 23 Iuliu Moldovan, 400348 Cluj-Napoca, Romania; 3 Department of Anatomic Pathology, University of Agricultural Sciences and Veterinary Medicine of Cluj-Napoca, 3-5 Calea Mănătur, 400372 Cluj-Napoca, Romania; 4 National Environmental Research Center, Sidi Amar Campus, BP 2024, 23005 Annaba, Algeria

**Keywords:** *Angiostrongylus vasorum*, Golden African wolf (*Canis lupaster*), Algeria

## Abstract

**Background:**

*Angiostrongylus vasorum*, commonly known as the “French heartworm,” is a nematode belonging to the Metastrongyloidea superfamily. This parasite was first identified in Toulouse, France in 1853 infecting the pulmonary arteries and the right side of the heart of a Pointer dog. Angiostrongylosis is an important infection due its severe clinical signs and potential for causing high morbidity and mortality in domestic dogs. This nematode has not been studied in Algeria. The aim of this study was investigate the presence of lungworms among different mammal species in a number of Algerian regions.

**Methods:**

Between February 2022 and September 2023, 47 road-killed animals were collected from six administrative units (departments) in Algeria. All carcasses underwent a full parasitological necropsy, and lung tissues were preserved in 10% buffered formalin and concentrated ethanol for further study. All collected samples were subjected to histological and PCR (cytochrome *c* oxidase subunit 1 gene) analyses for lungworm identification.

**Results:**

Histological examination revealed the presence of nematode eggs and larvae in the alveolar space and chronic obstructive vascular changes were detected in a single golden African wolf (*Canis lupaster*) collected from the department of Constantine. First-stage larvae were collected and morphologically identified as *Angiostrongylus* spp. The molecular identification confirmed the presence of *A. vasorum*. All other animals tested were negative for lungworms.

**Conclusions:**

To the best of our knowledge, this is the first report of *A. vasorum* infection in an African golden wolf (*Canis lupaster*). We report a new host association, highlighting the importance of further studies to update the geographical distribution of *A. vasorum* and its epidemiology across Algeria.

**Graphical Abstract:**

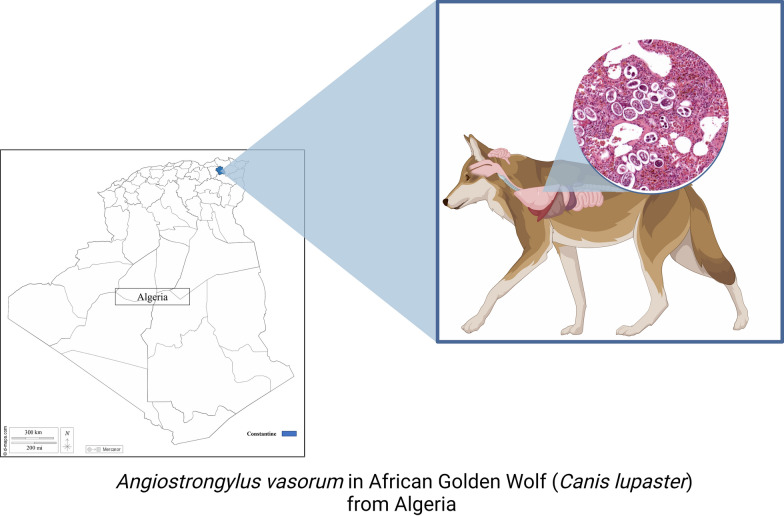

## Background

*Angiostrongylus vasorum*, commonly known as the “French heartworm”, was first identified in Toulouse, France by Serres in 1853 infecting the pulmonary artery and the right side of the heart of a Pointer dog [1]. Since then, the parasite has been described across a wide range of countries, in Europe, the Americas and Africa [2]. In the last two decades, the parasite gained much interest among researchers due to the severe clinical features and acute course of the infection in domestic dogs and its rapid expansion in Europe [3]. *Angiostrongylus vasorum* is the causative agent of angiostrongylosis in canids, which can present as various clinical signs, ranging from subclinical to lethal, with the most commonly observed symptoms including breathing difficulty, heart insufficiency, coagulation disorder, anorexia and decreased physical endurance. Clinical neurological signs are also common and can be manifested as ataxia, paresis, paralysis or seizures [4–6]. Unusual localizations of adult nematodes have been reported in the anterior chamber of the eye [7, 8], femoral artery [9], pericardial sac and urinary bladder [10], and larval stages have been identified in the brain [11]**,** diaphragm, pancreas, liver, and skin [10]**.**

This nematode has an indirect life-cycle using gastropods (snails and slugs) as intermediate hosts, while small vertebrates can act as paratenic hosts [6, 12–14]. The domestic chicken *Gallus domesticus* was shown to be a potentially suitable paratenic host in studies involving artificial infections [15]. The primary transmission route of *A. vasorum* is through the ingestion of third-stage larvae (L3) by the intermediate host (intentionally or accidentally) and/or a paratenic host. Infection can occur when the final host consumes food contaminated with infective intermediate hosts [4, 16] or water contaminated with L3 [17]**.** There is also the possibility of infection through the consumption of free L3 released in snail slime [17, 18]**.**

Among wild animals, wild foxes (*Vulpes vulpes*) commonly serve as the typical definitive hosts for *A. vasorum* [19], although there have been multiple reports of other wild canid definitive host species, such as the crab-eating fox (*Cerdocyon thous*), hoary fox (*Lycalopex vetulus*), coyote (*Canis latrans*), golden jackal (*Canis aureus*), gray wolf (*Canis lupus*) and raccoon dog (*Nyctereutes procyonoides*) [4, 12, 20–24]. Non-canid wild captive animals like the red panda (*Ailurus fulgens fulgens*) [25] and meerkats (*Suricata suricatta*) [26] have also been confirmed as definitive hosts. All of these hosts have the potential to act as reservoirs of *A. vasorum* for the infection for domestic dogs (*Canis familiaris*) [4, 27] and cats (*Felis catus*) [28]**.**

In Africa, the first *A. vasorum* infection was documented in Uganda in 1972, in five necropsied domestic dogs [29]; more recently, a potential autochthonous case in a 6-month-old asymptomatic dog was reported in Morocco based on the morphology of first-stage larvae (L1) [30].

There are 100 mammalian species in Algeria, belonging to 37 families and 11 orders, among which carnivores account for 21 species and seven families [31]. The diets of these species are mainly obtained by scavenging and/or predation, making these animals susceptible to food-borne parasitic infections [32]. Considering the abundant wild fauna in Algeria and the occurrence of the parasite in other northern African countries, the aim of this study was to investigate the presence of *A. vasorum* in animal hosts in Algeria.

## Methods

**Fig. 1:**
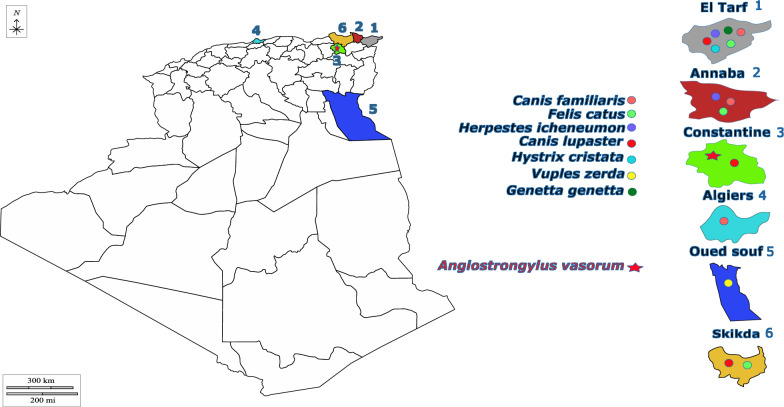
Map showing the geographical locations (departments) of the tested animals and the *Angiostrongylus vasorum*-positive animal

Between February 2022 and September 2023, the carcasses of 47 road-killed animals belonging to the orders Carnivora (11 *Canis familiaris*, 9 *Canis lupaster*, 6 *Felis catus*, 1 *Genetta genetta*, 3 *Herpestes ichneumon*, 3 *Vulpes vulpes* and 13 *Vulpes zerda*) and Rodentia (1 *Hystrix cristata*) were collected from 11 localities and six administrative units (departments) in Algeria (Table 1; Fig. 1) and transported to the National Research Center (CRE) in Annaba according to national governmental provisions. Prior to examination, the carcasses were entirely enclosed in a plastic bag (with identification) for health safety reasons and stored at - 20 °C. An extensive parasitological necropsy was performed on each carcass, and comprehensive data on each animal’s age and sexual maturity were collected according to [33, 34]. The entire cardio-respiratory system was removed from all animals, and the trachea, bronchi and bronchioles as well as the pulmonary arteries and the heart chambers were longitudinally opened and carefully examined for parasites using a stereomicroscope as described in [35]. During necropsy, macroscopic pictures of the lungs were taken where lesions were observed, samples of lung tissue were collected from each animal and preserved in both 10% buffered-formalin and concentrated ethanol separately. The collected samples were then legally transported to the Faculty of Veterinary Medicine (University of Agriculture and Veterinary Medicine) of Cluj-Napoca for molecular and histological analyses.

**Table 1:** Prevalence of lungworms in various animal species across different departments and localities

Department	Locality	Animal species	Number of examined animals	Animals positive for lungworms (*N*, %)
El Tarf	Ain kerma	*Canis familiaris*	6 (3 M, 3 F)	0
		*Canis lupaster*	1 M	0
		*Herpestes ichneumon*	1 M	0
	Asfour	*Canis lupaster*	1 F	0
	Bouhadjar	*Hystrix cristata*	1 M	0
		*Canis lupaster*	1 M	0
		*Vulpes vulpes*	2 (1 M, 1 F)	0
	Drean	*Felis catus*	1 M	0
		*Herpestes ichneumon*	1 M	0
		*Canis fammiliaris*	1 F	0
	Zitouna	*Genetta genetta*	1 F	0
		*Canis lupaster*	1 F	0
Annaba	Annaba	*Canis familiaris*	1 F	0
		*Canis lupaster*	2 (1 M, 1 F)	0
		*Felis catus*	4 (1 M, 3 F)	0
		*Herpestes ichneumon*	1 M	0
	Seraidi	*Canis Lupaster*	1 M	0
Constantine	Constantine	*Canis lupaster*	1 M	*Angiostrongylus vasorum* (1, 2.13%)
		*Vulpes vulpes*	1 F	0
Skikda	Skikda	*Felis catus*	1 M	0
		*Canis lupaster*	1 M	0
Algiers	Staoueli	*Canis familiaris*	2 (1 M, 1F)	0
Oued Souf	Oued souf	*Vulpes zerda*	13 (7 M, 6F)	0
Total			**47** **(24 M, 23F)**	**1, 2.13%**

*F* Female,* M* male

The formalin-fixed lung tissue samples were trimmed and embedded in paraffin wax, according to standard protocols, following which 2- to 3-μm-thick sections were obtained and stained with hematoxylin and eosin (H&E). The histological assessment was performed using a light Olympus BX-41 microscope equipped with an Olympus SP 350 digital camera (Olympus Corp., Tokyo, Japan). The photomicrographs were taken using the Stream Basic imaging software (Olympus Corp.). When the histological examination showed a parasitic infection, the entire quantity of formalin in which lungs were conserved was placed in a 15-ml conical centrifuge tube and centrifuged (13,000*g*); the obtained sediment was examined under a stereomicroscope. One drop of Lugol solution was added directly onto the slide for a better view of larvae while taking pictures. Genomic DNA was isolated from small lung sections conserved in concentrated ethanol from all animals using a commercial kit (Isolate II Genomic DNA kit; Meridian Bioscience, London, UK) according to the manufacturer's instructions. DNA was also isolated from a pool of larvae collected from the ethanol solution in which the African wolf lung tissues were conserved. A fragment of the mitochondrial cytochrome* c* oxidase subunit 1 (cox1, approx. 700 bp) gene was then amplified by conventional PCR using the universal primers LCO1490/HCO2198, according to protocols reported in the literature [36]. The obtained PCR products were visualized following electrophoresis in 2% agarose gels stained with EcoSafe nucleic acid staining solution (Pacific Image Electronics, Taiwan), and their molecular weight was assessed by comparison to a molecular marker (HyperLadder™ 100 bp, meridian Bioscience, UK). The obtained band was excised from the gel, purified on a silica membrane spin column (Gel/PCR DNA Fragments Kit; Geneaid Biotech, New Taipei City, Taiwan), and sequenced bidirectionally using an external service (performed by Macrogen Europe B.V., Amsterdam, the Netherlands). The sequences were assembled using Geneious software (Biomatters Ltd, Auckland, New Zealand) and compared to other sequences available in the NCBI GenBank® database by Basic Local Alignment Search Tool (BLAST) analysis.

## Results

**Fig. 2:**
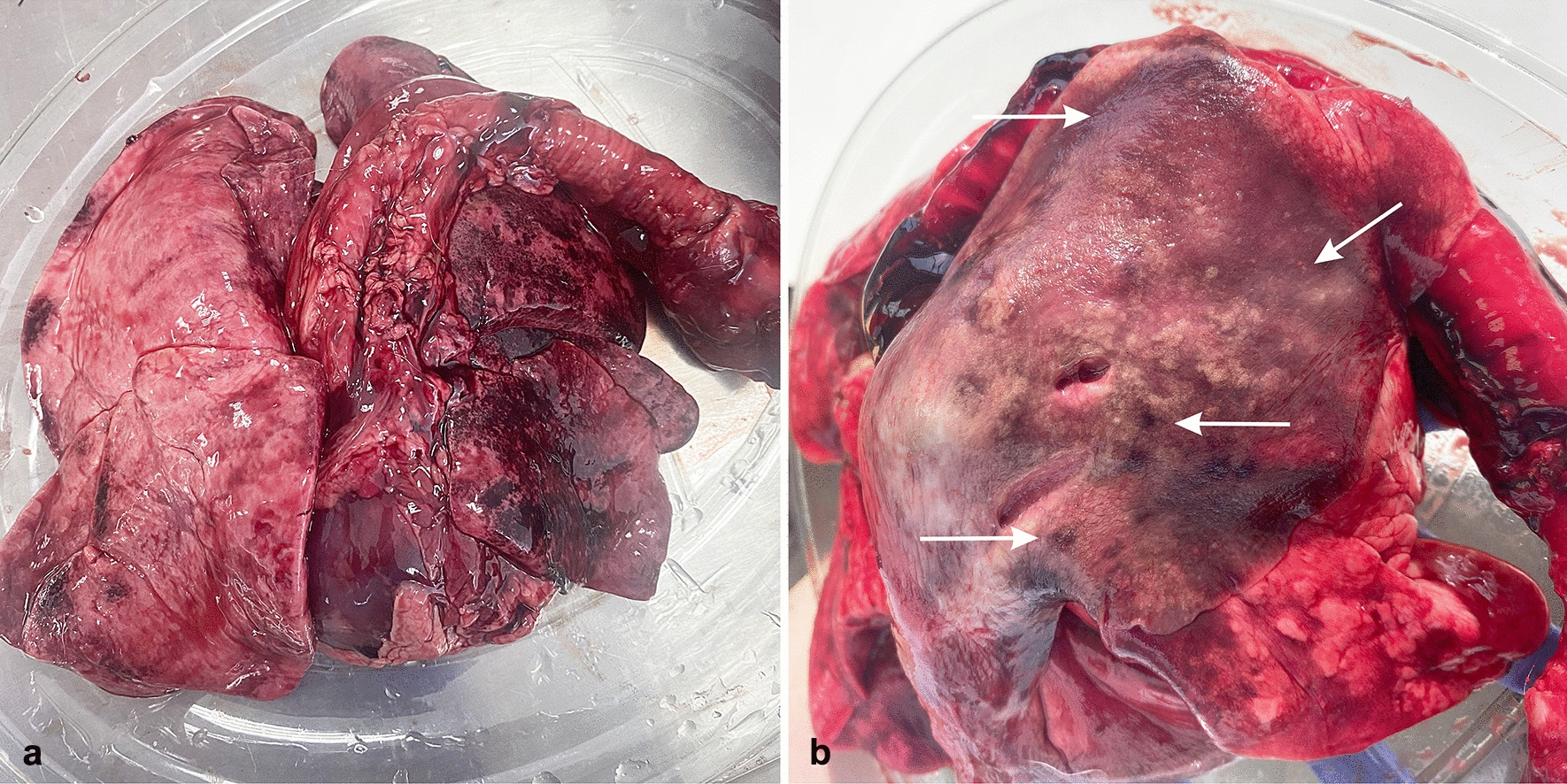
Gross evaluation of the lungs of an African golden wolf (*Canis lupaster*) with suspected *Angiostrongylus vasorum* infection. **a**, **b** Pulmonary parenchyma showing multifocal to coalescing dark-red areas (arrows) consistent with verminous pneumonia and severe alteration of blood vessels

**Fig. 3:**
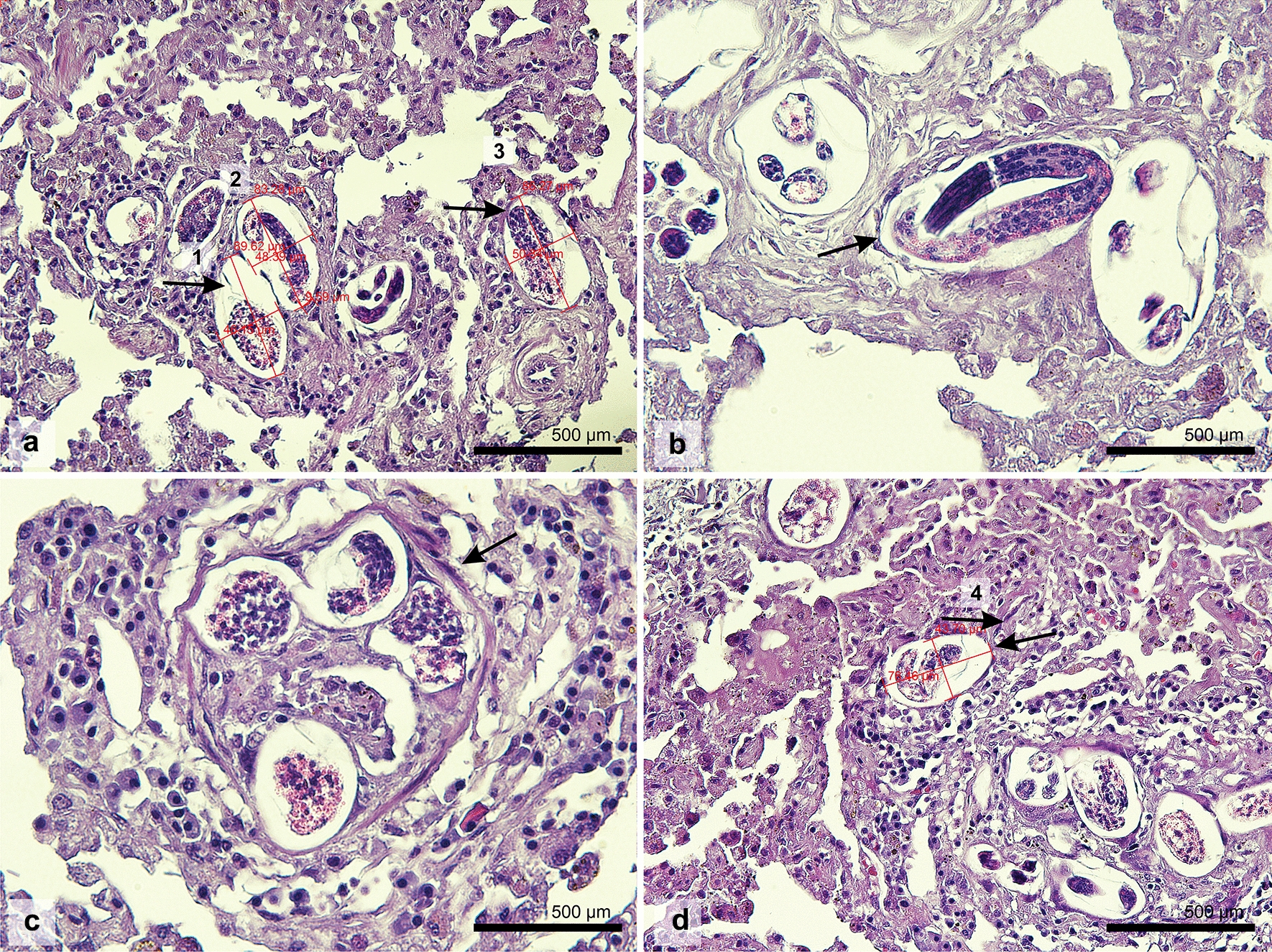
Microscopical findings of pulmonary angiostrongylosis in an African golden wolf (*Canis lupaster*) with suspected *Angiostrongylus vasorum* infection. **a**–**d** The pulmonary interstitium is moderately expanded by numerous inflammatory nodules centered on parasitic eggs and larvae (arrows)

**Fig. 4:**
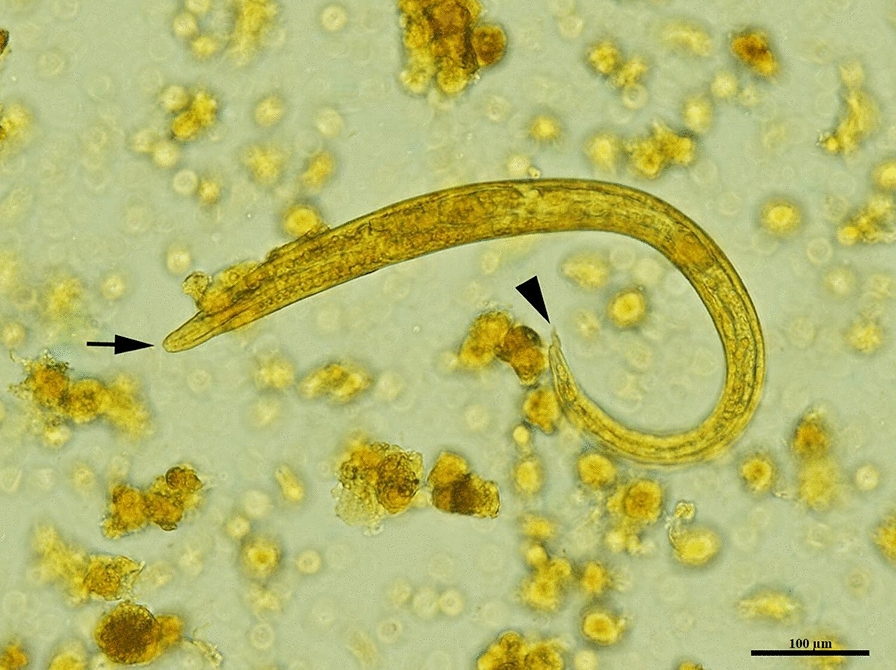
First-stage larva of *Angiostrongylus vasorum* collected from the lungs. Note the median cranial oral opening and the kinked tail with a dorsal spine and a notch

At the time of the necropsy, adult lungworms were not detected in any of the examined animals. In one single African golden wolf (*Canis lupaster*) collected from Constantine department (36°21′N 6°36′E), the pulmonary parenchyma showed moderate congestion and edema, multifocal dark-red and variably-sized areas of densification, compatible with foci of ischemic necrosis, severe hemorrhage and interstitial verminous pneumonia (Fig. 2a, b). The lesions were mainly located at the periphery of the lung parenchyma. No adult parasites were observed within the right ventricle and pulmonary arteries during the macroscopical and microscopical examination. The lungs of all other examined animals had no serious macroscopic lesions. In the African golden wolf with pulmonary lesions, histological examination revealed numerous transverse to longitudinal sections of nematode larvae and free eggs in the alveolar spaces and interstitium, associated with a mixed inflammatory reaction composed mainly of macrophages, including binucleated and multinucleated cells, plasma cells, small lymphocytes and a few eosinophils. The larvae were approximately 20–30 µm in width with a thin basophilic cuticle and numerous deep basophilic, internal nuclei. The eggs were round to ovoid, thin-walled and approximately 50–60 µm in diameter; they contained either a larva or a morula. Additional findings included arterial thrombosis, proliferative endarteritis with recanalization, pulmonary necrosis, hemorrhage, edema, interstitial fibrosis, type II pneumocyte hyperplasia and small aggregates of hemosiderin-laden macrophages (Fig. 3a–d). No histological changes related to lungworms were noted in the pulmonary parenchyma collected from the other animals. Larvae recovered from the sediment of the centrifuged solutions were morphologically consistent with *Angiostrongylus* spp. larvae, having a terminal oral opening and a kinked tail with a spine and a notch (Fig. 4).

The BLAST analysis revealed 99.17–100% nucleotide identity to numerous other *A. vasorum* isolates from dogs and foxes registered in Europe (e.g. GenBank Accession Numbers: OQ210698, GQ982791, GQ982874). The sequence was deposited in GenBank under Accession Number PP872515.

All other animals were also negative for PCR as well.

## Discussion

In the present study, we demonstrated the presence of *A. vasorum* in Algeria, provided the first molecular confirmation of this species in Africa, and report a new host-parasite association. The finding of *A. vasorum* may indicate the continuous geographical expansion of this parasite to new areas that were previously not considered endemic, as recorded also in Morocco [30]. Interestingly, although the first report of *A. vasorum* in Africa dates back to > 50 years ago, when it was identified in domestic dogs from Uganda [29], the lack of more recent reports could be attributed to the lack of awareness or lack of interest among veterinarians and dog owners regarding this potentially fatal disease, as noted in a previous study [4], or, more likely, to the sporadic occurrence of *A. vasorum* in Africa. Another hypothesis would be that *A. vasorum* was introduced into northern Africa more recently. In the more recent report from Morocco, *A. vasorum* was identified in a symptomatic domestic dog (*C. familiaris*) from Rabat city using morphological identification of L1 collected from feces. The occurrence of *A. vasorum* is related to several factors, both biotic (humidity, temperature) and abiotic (intermediate, paratenic and final hosts). Numerous gastropod species have been reported in the study area from Algeria where the *A. vasorum*-positive carcass was collected [37]. However, the specificity of *A. vasorum* for the intermediate host is broad, and many snails and slugs can host the larvae for their development [27]. In endemic areas in Europe, the emergence of this parasite could be linked to various factors, such as climate change, urbanization of the red fox and dog movement/transport [38].

The present finding underlines the importance of complementary diagnostic methods for the detection and identification of lungworms, as has been previously recommended [35]. Once again, histology proved to be a key method for the detection of *A. vasorum* infection [39]. Explanations for not finding adult nematodes during the necropsy likely include a low number of worms or the lack of use of additional methods, such as the artificial digestion technique. Examination of feces using the larval concentration method may also have revealed some larvae although the carcass was frozen [40]. The absence of infection in the carcasses of the other animal species examined might be correlated to the low number of examined animals, their food habits, their unsuitability as hosts, the recent introduction of the parasite to Algeria or the patchy distribution of the parasite.

There are many stray and free-roaming dogs in Algeria [41] that share the same lifestyle (scavenging) as other canids, such as the African golden wolf, and these could be easily infected with *A. vasorum*. Therefore, further studies are required for a better understanding of the epidemiological scenario of *A. vasorum* and to determine the potential life-cycle pattern of *A. vasorum* in Algeria.

## Conclusions

This new host and geographical record of *A. vasorum* expands current knowledge on this clinically important parasite, highlighting the importance of studies in wild carnivores from areas where the investigations on their parasitic fauna have been historically limited. Based on these results, we also advocate the use of complemental diagnostic techniques when examining dead animals. Additionally, due to the possible severe evolution of the infection in domestic dogs, awareness among local veterinarians should be raised for including infections by *A. vasorum* on differential diagnosis, especially in symptomatic animals.

## Data Availability

No datasets were generated or analyzed during the current study.
